# Supplementary material for: Urinary Volatile Organic Compound Analysis for the Diagnosis of Cancer: A Systematic Literature Review and Quality Assessment
Source: Metabolites. 2020 Dec 29;11(1):17. doi: 10.3390/metabo11010017 (PMC7824454; doi:10.3390/metabo11010017)
Supplement: Supplementary file 1 [file metabolites-11-00017-s001.pdf]

Review

Urinary volatile organic compound analysis for the diagnosis of cancer: a systematic literature review and quality assessment

Qing Wen [1], Piers Boshier [1], Antonis Myridakis [1], Ilaria Belluomo [1], and George B. Hanna [1,*]

[1] Department of Surgery and Cancer, Imperial College London, St Mary's Hospital, London, W2 1NY, UK
* Correspondence: g.hanna@imperial.ac.uk; Tel.: +44-(0)207-886-2125

Search Strategy

Database: Embase Classic+Embase <1947 to 2019 December 13>
Search Strategy:
----
1 Volatile Organic Compounds/ or volatile organic compound*.mp. (20445)
2 biomarkers.mp. or BIOMARKERS/ (385783)
3 Metabolomics/ or metabo*omics.mp. (35735)
4 Magnetic Resonance Spectroscopy/ or magnetic resonance spectrometry.mp. (100046)
5 mass spectrometry.mp. or Mass Spectrometry/ (463935)
6 metabolic profiling.mp. (4883)
7 metabotyping.mp. (71)
8 urine.mp. or URINE/ (564820)
9 1 or 2 (405092)
10 3 or 4 or 5 or 6 or 7 (569457)
11 8 and 9 and 10 (5080)
12 prostate.mp. or PROSTATE/ (347922)
13 prosta*.mp. (583298)
14 gastric.mp. or GASTRIC/ (369992)
15 gastro*.mp. (894775)
16 esophageal.mp. or ESOPHAGEAL/ (149069)
17 esophago*.mp. (53403)
18 colorectal.mp. or COLORECTAL/ (261334)
19 rectal*.mp. (153592)
20 bowel.mp. or BOWER/ (249315)
21 intestinal.mp. or INTESTINAL/ (386822)
22 intestin*.mp. (799633)
23 pancreas.mp. or PANCREAS/ (394885)
24 pancrea*.mp. (520745)
25 carcinoma.mp. or CARCINOMA/ (1218118)
26 ca*.mp. (24378921)
27 12 or 13 (583298)
28 14 or 15 (1143350)
29 16 or 17 (180363)
30 18 or 19 or 20 or 21 or 22 (1231013)
31 23 or 24 (520745)
32 25 or 26 (24378921)
33 27 or 28 or 29 or 30 or 31 (3084057)
34 32 and 33 (2400354)
35 11 and 34 (689)
36 limit 35 to english language (676)
37 limit 36 to human (571)
****

Database: Ovid MEDLINE(R) ALL <1946 to December 13, 2019>
Search Strategy:
----
1 Volatile Organic Compounds/ or volatile organic compound*.mp. (14685)
2 biomarkers.mp. or BIOMARKERS/ (491461)
3 Metabolomics/ or metabo*omics.mp. (25507)
4 Magnetic Resonance Spectroscopy/ or magnetic resonance spectrometry.mp. (147500)
5 mass spectrometry.mp. or Mass Spectrometry/ (309505)
6 metabolic profiling.mp. (3623)
7 metabotyping.mp. (55)
8 urine.mp. or URINE/ (357244)
9 1 or 2 (505235)
10 3 or 4 or 5 or 6 or 7 (454801)
11 8 and 9 and 10 (4492)
12 prostate.mp. or PROSTATE/ (189208)
13 prosta*.mp. (364594)
14 gastric.mp. or GASTRIC/ (287312)
15 gastro*.mp. (522330)
16 esophageal.mp. or ESOPHAGEAL/ (137920)
17 esophago*.mp. (37648)
18 colorectal.mp. or COLORECTAL/ (154456)
19 rectal*.mp. (114296)
20 bowel.mp. or BOWER/ (151056)
21 intestinal.mp. or INTESTINAL/ (403809)
22 intestin*.mp. (499655)
23 pancreas.mp. or PANCREAS/ (137239)
24 pancrea*.mp. (325641)
25 carcinoma.mp. or CARCINOMA/ (805969)
26 ca*.mp. (18137476)
27 12 or 13 (364594)
28 14 or 15 (714689)
29 16 or 17 (150433)
30 18 or 19 or 20 or 21 or 22 (799204)
31 23 or 24 (325641)
32 25 or 26 (18137476)
33 27 or 28 or 29 or 30 or 31 (2026101)
34 32 and 33 (1468790)
35 11 and 34 (449)
36 limit 35 to english language (435)
37 limit 36 to human (315)
****

PRISMA Checklist

Section/topic	#	Checklist item	Reported on page #
**TITLE**			
Title	1	Identify the report as a systematic review, meta-analysis, or both.	1
**ABSTRACT**			
Structured summary	2	Provide a structured summary including, as applicable: background; objectives; data sources; study eligibility criteria, participants, and interventions; study appraisal and synthesis methods; results; limitations; conclusions and implications of key findings; systematic review registration number.	1; no registration number
**INTRODUCTION**			
Rationale	3	Describe the rationale for the review in the context of what is already known.	1-2
Objectives	4	Provide an explicit statement of questions being addressed with reference to participants, interventions, comparisons, outcomes, and study design (PICOS).	2
**METHODS**			
Protocol and registration	5	Indicate if a review protocol exists, if and where it can be accessed (e.g., Web address), and, if available, provide registration information including registration number.	13
Eligibility criteria	6	Specify study characteristics (e.g., PICOS, length of follow-up) and report characteristics (e.g., years considered, language, publication status) used as criteria for eligibility, giving rationale.	13-14
Information sources	7	Describe all information sources (e.g., databases with dates of coverage, contact with study authors to identify additional studies) in the search and date last searched.	13
Search	8	Present full electronic search strategy for at least one database, including any limits used, such that it could be repeated.	13-14, Supplementary
Study selection	9	State the process for selecting studies (i.e., screening, eligibility, included in systematic review, and, if applicable, included in the meta-analysis).	13-14
Data collection process	10	Describe method of data extraction from reports (e.g., piloted forms, independently, in duplicate) and any processes for obtaining and confirming data from investigators.	13-14

Data items	11	List and define all variables for which data were sought (e.g., PICOS, funding sources) and any assumptions and simplifications made.	14
Risk of bias in individual studies	12	Describe methods used for assessing risk of bias of individual studies (including specification of whether this was done at the study or outcome level), and how this information is to be used in any data synthesis.	13-14
Summary measures	13	State the principal summary measures (e.g., risk ratio, difference in means).	14
Synthesis of results	14	Describe the methods of handling data and combining results of studies, if done, including measures of consistency (e.g., I2) for each meta-analysis.	N/A
Risk of bias across studies	15	Specify any assessment of risk of bias that may affect the cumulative evidence (e.g., publication bias, selective reporting within studies).	13-14
Additional analyses	16	Describe methods of additional analyses (e.g., sensitivity or subgroup analyses, meta-regression), if done, indicating which were pre-specified.	N/A
**RESULTS**			
Study selection	17	Give numbers of studies screened, assessed for eligibility, and included in the review, with reasons for exclusions at each stage, ideally with a flow diagram.	2, Figure 1
Study characteristics	18	For each study, present characteristics for which data were extracted (e.g., study size, PICOS, follow-up period) and provide the citations.	2, Table 1
Risk of bias within studies	19	Present data on risk of bias of each study and, if available, any outcome level assessment (see item 12).	6, Table 2, Table S1
Results of individual studies	20	For all outcomes considered (benefits or harms), present, for each study: (a) simple summary data for each intervention group (b) effect estimates and confidence intervals, ideally with a forest plot.	8-10, Figure 2, 3 and 4, Table 3, Table S2
Synthesis of results	21	Present results of each meta-analysis done, including confidence intervals and measures of consistency.	N/A
Risk of bias across studies	22	Present results of any assessment of risk of bias across studies (see Item 15).	6, Table 2, Table S1
Additional analysis	23	Give results of additional analyses, if done (e.g., sensitivity or subgroup analyses, meta-regression [see Item 16]).	N/A
**DISCUSSION**			
Summary of evidence	24	Summarize the main findings including the strength of evidence for each main outcome; consider their relevance to key groups (e.g., healthcare providers, users, and policy makers).	11-13, Table 4
Limitations	25	Discuss limitations at study and outcome level (e.g., risk of bias), and at review-level (e.g., incomplete retrieval of identified research, reporting bias).	13

Conclusions	26	Provide a general interpretation of the results in the context of other evidence, and implications for future research.	13
**FUNDING**			
Funding	27	Describe sources of funding for the systematic review and other support (e.g., supply of data); role of funders for the systematic review.	15

Supplementary Materials

$$\frac{1/(\text{Total number of VOCs identified in each study})}{\text{Total number of VOCs identified in each cancer type}} \times \begin{array}{l}\text{Total number of studies}\\ \text{that identified this VOC}\end{array}$$

Figure S1. Equation for weighted means of each identified volatile organic compound (VOC).

Table S1. Quality assessment of metabolic metadata based on CAWG-MSI guidelines.

	Sample preparation								Experimental analysis					Instrumental performance					Method validation													Metabolite identification										Data pre-processing		Metabolite identification
Author	Biological replicates	Biofluid harvesting	Storage condition	Extraction method	Extract concentration, dilution, resolubilisation process	Extract enrichment	Extract cleanup	Extract storage and/or relocation	Instrument description	Auto-injector	Separation column	Technique specific sample preparation	Separation parameters	Instrument description	Sample introduction & delivery	Ionisation source	Mass analyser description and acquisition mode	Data acquisition parameters	m/z calibration standard	Accuracy of m/z calibration	Mass resolution	Ion source optimisation parameters	Chromatographic resolution	QC samples	Internal Standards used	Accuracy of Internal standard	Precision of internal standard	Retention time markers	Accuracy and precision for replicated analyses	Accuracy and precision for validation samples	Cycles per column/injector/septum/blank	Relative quantification			Absolute quantification							Post-acquisition data pre-processing		
																																Description, quantifier of IS metabolite	Method for assessing instrument	Replicate analyses	Calibration curves for each metabolite	Range of standards used	Quantification of method accuracy	Quantification of method precision	LLOQ	LLOD	Recovery/ stability	Data file format/ conversion methods	Data-preprocessing methods	Level

Lima A.R. 2019	Y	Y	Y	Y	N	Y	N	Y	Y	Y	Y	Y	Y	Y	Y	Y	Y	Y	N	NA	N	Y	N	Y	Y	N	N	N	N	Y	N	Y	Y	N								N	Y	2
Struck-Lewicka W. 2014	Y	Y	Y	Y	Y	N	N	N	Y	N	Y	Y	Y	Y	N	Y	Y	Y	N	NA	N	Y	N	Y	Y	N	N	Y	N	N	N	Y	Y	N								N	Y	2
Gao Q. 2019	Y	Y	Y	Y	N	Y	N	Y	Y	N	Y	Y	Y	Y	N	Y	Y	Y	N	NA	N	N	N	N	Y	N	N	N	N	Y	N	Y	N	N								N	Y	2
Jimenez-Pacheco A. 2018	Y	Y	Y	Y	N	Y	N	Y	Y	Y	Y	N	Y	Y	N	Y	Y	Y	N	NA	N	N	N	N	N	NA	NA	N	N	N	N	N	N	N								N	N	2
Khalid T. 2015	Y	Y	Y	Y	N	Y	N	Y	Y	N	Y	N	Y	Y	N	Y	Y	Y	N	NA	N	N	N	N	N	NA	NA	N	N	N	N	N	Y	Y								N	N	2
Spanel P. 1999	Y	Y	Y	N	N	N	N	N	Y	N	N	N	N	Y	Y	Y	Y	Y	N	NA	N	N	N	N	N	NA	NA	N	N	N	N				N	N	N	N	N	N	N	N	N	1
Chen Y. 2016	Y	Y	Y	Y	Y	N	N	N	Y	N	Y	Y	Y	Y	N	Y	Y	Y	N	NA	N	N	N	N	Y	N	N	N	N	Y	N				Y	Y	Y	Y	Y	Y	Y	N	N	1
Navaneethan U. 2015	Y	Y	Y	Y	N	N	N	N	N	N	N	N	N	Y	Y	Y	Y	Y	N	NA	N	N	N	N	N	NA	NA	N	Y	N	N				N	N	N	N	N	N	Y	N	Y	2
Panebianco C 2017	Y	Y	Y	Y	N	Y	N	N	Y	Y	Y	N	Y	Y	Y	N	N	N	N	NA	N	N	N	N	N	NA	NA	N	N	N	N	N	Y	N								N	Y	2
Arasaradnam RP 2014	Y	Y	Y	Y	N	N	N	N	Y	Y	Y	N	Y	Y	N	Y	Y	Y	N	NA	N	Y	N	N	N	NA	NA	N	N	N	N	N	Y	N								N	Y	NA
Huang J. 2013	Y	Y	Y	N	N	N	N	N	N	N	N	N	N	Y	Y	Y	Y	Y	N	NA	N	N	N	N	N	NA	NA	N	N	N	N				N	N	N	Y	N	N	N	N	N	2
Rozhentsov A.A. 2014	Y	N	Y	Y	N	Y	N	Y	N	N	N	N	Y	N	N	N	N	N	N	NA	N	N	N	N	N	NA	NA	N	N	N	N				N	N	N	N	N	N	N	N	N	NA
Silva C.L. 2011	Y	Y	Y	Y	N	Y	N	Y	Y	N	Y	N	Y	Y	Y	Y	Y	Y	N	NA	N	Y	N	N	N	NA	NA	N	N	N	N	N	N	N								N	N	2

Y: yes, N: no, NA: not applicable. QC: quality control; IS: internal standard; LLOQ: lower limits of quantification; LLOD: lower limits of detection

Table S2. List of biomarkers noted to be significantly increased and/or decreased in cancers and associated metabolic pathway.

Compound name	Chemical Classes	Metabolic pathway	Prostate Cancer	Gastrointestinal Cancer
Ethanol	Alcohol	Glycolysis / Gluconeogenesis		↓
Acetaldehyde	Aldehyde	Pyruvate metabolism; Glycolysis / Gluconeogenesis; Glycerophospholipid metabolism		↑
Phenylacetic acid	Aromatic compound	Phenylalanine metabolism		↑
Butyric acid	Fatty acid	Butanoate metabolism	↓	
Methylglyoxal	Ketoaldehyde	Glycine, serine and threonine metabolism; Pyruvate metabolism	↓	
Acetic acid		Glyoxylate and dicarboxylate metabolism; Pyruvate metabolism; Glycolysis / Gluconeogenesis	↓	↑
Hydrogen sulfide	Organic acid	Sulfur metabolism		↑
Propionic acid		Propanoate metabolism	↓	
